# Feasibility and acceptability of implementing an evidence-based ESCALATION system for paediatric clinical deterioration

**DOI:** 10.1038/s41390-024-03459-y

**Published:** 2024-08-13

**Authors:** Fenella J. Gill, Alannah Cooper, Pania Falconer, Scott Stokes, Alison Roberts, Matthew Szabo, Gavin D. Leslie

**Affiliations:** 1 School of Nursing, Faculty of Health Sciences, Curtin University, Perth, WA Australia; 2 Nursing Research, Perth Children’s Hospital, Child & Adolescent Health Services, Nedlands, WA Australia; 3 Nursing and Midwifery Research Unit, South Metropolitan Health Service, Murdoch, WA Australia; 4 Nursing Research, St John of God Healthcare, Subiaco, WA Australia; 5 Clinical Nursing Research Unit, Royal Perth Hospital, Perth, WA Australia; 6 Kimberley Regional Paediatric Service, Broome Hospital, Western Australia Country Health Service, Kimberley, WA Australia; 7 National School of Nursing and Midwifery, University of Notre Dame Australia, Broome, WA Australia; 8 Department of Endocrinology and Diabetes, Child and Adolescent Health Service, Nedlands, WA Australia; 9 Children’s Diabetes Centre, Telethon Kids Institute, Nedlands, WA Australia

## Abstract

**Background:**

The ESCALATION system is a novel paediatric Early Warning System that incorporates family involvement and sepsis recognition. This study aimed to assess the feasibility and iteratively refine the ESCALATION system in a variety of hospital settings in preparation for full-service implementation.

**Methods:**

A series of four multi-methods studies using an Implementation Science and co-design approach were conducted. We examined concepts of implementation, context, and mechanisms of action across a variety of hospitals. Data collected included practice and chart audits, surveys (health professionals), interviews (families) and focus groups (health professionals). Quantitative data were analysed descriptively with qualitative findings assessed by content analysis or thematic analysis.

**Results:**

There were 650 audits (Study I–IV), 205 health professional survey responses (Study I), 154 health professionals participated in focus groups (Study II–IV), 13 parents of hospitalised children interviewed (Study I), and 107 parents reported their involvement in the ESCALATION system (Study III–IV). Each of the studies further refined and confirmed the feasibility, specifically the components of family involvement and the sepsis recognition pathway.

**Conclusion:**

The Implementation Science evaluation of the ESCALATION system resulted in a uniform approach that was feasible and acceptable to users and appropriate for full-service implementation.

**Impact:**

- This series of four studies used a co-production approach built on the Medical Research Council framework to understand feasibility and acceptability of an intervention to improve recognition and response to clinical deterioration in children to the point of full-service implementation.
- We have reported a detailed, systematic approach to assessing feasibility and acceptability of a complex intervention using established methodologies for whole of health system implementation.
- The ESCALATION System is an evidence based paediatric early warning system that is a highly refined, well accepted and accommodates a health system that has substantial contextual variation.

## Introduction

Delays in recognition and response to clinical deterioration are causes of sub-optimal care for hospitalised children. Early Warning Systems (EWS) have been widely implemented in hospitals to promote timely detection of changes in a child’s condition that may forewarn of clinical deterioration.^1^ Early Warning System components include observation and response charts featuring graphical information to track trends of vital signs, specified thresholds indicating increasing variance from acceptable ranges to trigger an escalation of care response, and escalation of care pathways for increasing urgency of response.

Despite widespread use of EWS there continue to be systemic failures including delays to detect and address acute clinical deterioration. Recent high-profile hospital incidents^2,3^ and coronial cases^4–8^ have noted serious adverse outcomes including death. Especially worrying in paediatric contexts are cases where families’ concerns about their child’s deteriorating health condition have gone unheard by health professionals and signs of clinical deterioration overlooked.^6,8,9^

EWS are complex interventions,^10^ in that they involve several interacting components, target a range of desired behaviours, require effective interactions between professional groups with specific expertise and skills across multiple healthcare settings with a variability of possible outcomes.^11^ Family activated escalation of care processes for clinical deterioration largely remain separate to EWS. There have been many reported barriers to implementation and effective use of processes for family involvement in escalation of care including families’ low levels of awareness, lack of confidence to raise concerns, or reservations about challenging health professionals.^12,13^ Health professionals’ beliefs and attitudes add additional complexity to family involvement in escalation of care.

The leading cause of unidentified deterioration preceding preventable childhood death and disability is sepsis.^14^ Approximately one third of sepsis deaths occur in previously healthy children and another third of survivors suffer long-term morbidity.^14^ Sepsis is preventable if detected and treated in a timely manner, yet delays in recognition and management of sepsis are common.^8,14^ International^15^ and Australian guidelines^16,17^ recommend use of tools for systematically screening for sepsis to promote early recognition, including identifying patients at risk of sepsis. Components missing from existing paediatric EWS are integrated family involvement and sepsis recognition pathways.

The ESCALATION system is an EWS designed for use with acutely unwell children in the largest state in Australia, covering an area of 2,529,875 square km, encompassing the entire western third of the country. Despite the immense size, it is one of the least densely populated states in the world, with a total population of 2.7 million most of which (92%), is concentrated in the south-west, with a large proportion of the state sparsely populated.^18^ The low population density, coupled with vast distances between healthcare facilities, creates unique challenges in healthcare delivery. The approach to assessing paediatric patients in Western Australia had been identified as inconsistent and inadequate.^19^ The ESCALATION system, developed specifically for the West Australian context, integrates both family involvement and sepsis recognition with escalation of care pathways developed to suit operational contexts, acknowledging complex interventions work best if tailored to local needs rather than being completely standardised.^10^

Best practice is to develop interventions systematically, with the ESCALATION development process being based on Hawkins et al.^20^ three-stage framework of (1) evidence review, stakeholder engagement and consultation, (2) co-production by researchers and stakeholders planning and developing the intervention, and (3) prototyping and testing the intervention.^19^ The resultant ESCALATION system incorporates a whole-system approach to early recognition of clinical deterioration and effective and timely escalation of care.^19^ The theoretical underpinning is key ESCALATION system components interact to support health professionals’ critical thinking and situational awareness to detect and respond to early signs of clinical deterioration.

Building on the initial ESCALATION system development,^19^ we report a series of studies based on an Implementation Science (IS) approach to examine the mechanisms of the intervention and how those mechanisms might influence or be influenced by the context.^21^ Our aim was to understand the feasibility of using the ESCALATION system in a variety of hospital settings and iteratively refine the system in preparation for state-wide service implementation.

## Methods

**Fig. 1:**
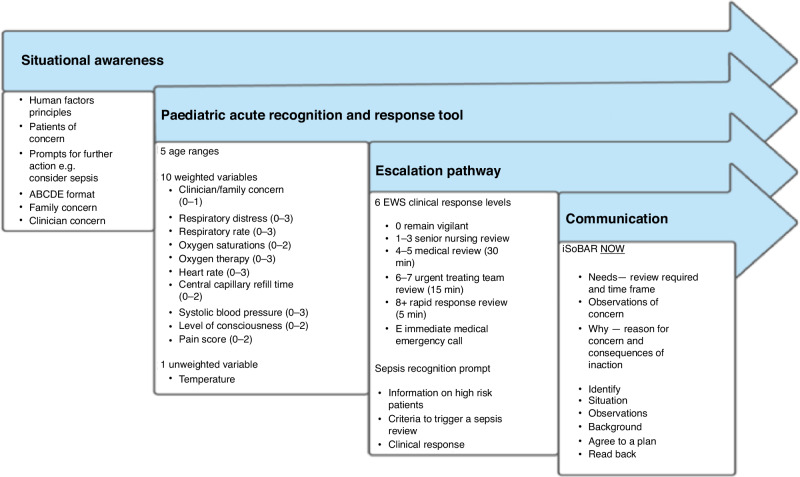
Components of the ESCALATION system. Adapted from Gill, FJ. et al.^19^ “Development of an evidence-based ESCALATION system for recognition and response to paediatric clinical deterioration.” Australian critical care 35(6): 668-676.

The Medical Research Council (MRC) framework for developing and evaluating complex interventions describes five research phases that encompass; developing, piloting and feasibility, evaluating, reporting through to implementation.^11^ This series of multi-methods studies, spanning 2019–2022, used a co-production approach and draws on the MRC framework^11^ recommendations that emphasise the importance of adequate intervention piloting and feasibility work to fully consider practical issues prior to implementation. Findings from each study informed subsequent studies to increase transferability across settings to the final system for full-service implementation Fig. 1.

The feasibility of the intervention and logistics for delivery in specific settings were examined through close monitoring of activities performed and users’ capability of carrying out the components of the intervention as planned.^22^ Acceptability was assessed through users’ attitude towards the intervention. Feasibility and acceptability indicators are reported using process evaluation concepts of -:

- *implementation* – what and how the intervention was delivered?
- *context* -what influenced it (the intervention)?
- *mechanisms of impact –* how does the delivered intervention produce change?^23^

The research co-production^24^ was a partnership between researchers and knowledge users enabled through a steering group of stakeholders (20 representatives from five health services, private hospitals, community health services, state pre-hospital emergency response service, state aeromedical emergency service, and two health consumers). Additionally, two health consumer advisory groups provided a family (seven members) and youth (six members) perspective throughout. Where focusing specifically on chart design, education, training and implementation, smaller groups of health professionals and consumers worked with the research team.^19^

**Fig. 2:**
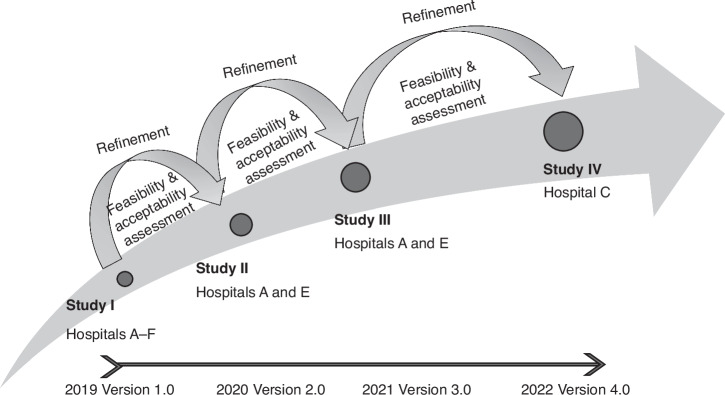
Study design series of 4 studies examining feasibility and acceptability across a range of settings.

Note as illustrated in Fig. 2 our series of studies involved six hospitals (A-F described in Table 1) and four versions of the ESCALATION system. In Study I (ESCALATION version 1.0) we examined System feasibility and acceptability across a range of settings. For Study II and Study III (ESCALATION versions 2.0–3.0), our focused assessments were System refinements in two different settings (regional hospital E and the children’s hospital A). In Study IV we further focused assessment at metropolitan hospital C to assess refinements and feasibility of version 4.0 ESCALATION pathway ensuring System readiness for implementation.

**Table 1:** Hospital characteristics

	Hospital A	Hospital B	Hospital C	Hospital D	Hospital E	Hospital F
Location	Metropolitan	Regional	Metropolitan	District	Regional	Metropolitan
Service	Specialist paediatric	Visiting paediatric service	Paediatric and adult services	No paediatric service	Paediatric and adult services	Paediatric and adult services
Public/private	Public	Public	Public	Public	Public	Public and private
ICU present	Paediatric ICU	No ICU	Adult ICU	No ICU	Adult ICU	Adult ICU
Year 2018 mean paediatric patients						
ED presentations (P)	P 70000	P 5800	P 26000	P 1750	P 5400	P 25000
Ward admissions (A)	A 30000	A 750	A 3200	A 150	A 850	A 1400
Existing paediatric EWS	Composite score	Single trigger	Composite score	Single trigger	Single trigger	Composite score

### Implementation – what and how the intervention was delivered?

To understand intervention feasibility we examined and refined:

- *Bedside audits* based on previously reported studies,^25,26^ tested for clarity with six ESCALATION charts (Online File: bedside audit study I). Audits were conducted after 8 weeks to examine the degree of fidelity (intervention delivered as intended) evident through recording of variables and early warning score, response and actions to escalate care. Each study audit results enabled provision of feedback to promote increased fidelity,
- *Implementation preparation* that involved multiple education and training approaches, iteratively refined and improved (Table 2).
  - A dedicated website (www.escalation.com.au) supported communication and sharing of education resources.
  - Workshops attended by “Champions” who were educators and other key nurses who volunteered to be ESCALATION leads. Workshops consisted of instruction, demonstration, discussion, case studies, clinical simulation and sharing of previous study findings. The feedback enabled Champions to target education strategies.
  - Information packs, posters, pamphlets and fact sheets supported the Champions to provide health professional and family education at their own hospitals. Study 1 evaluation resulted in setting a minimum target of 80% of staff at all sites to receive education and training in preparation for Study II.
  - For Study II videos were developed and used at training sessions to provide instruction and demonstration using the ESCALATION System in simulated clinical scenarios. Further refinement in preparation for Study III involved developing an education package hosted on an online learning management platform. The videos and PowerPoint™ presentations were embedded in the package and covered key components including involving family in assessment documenting variables, calculating the early warning score, escalation of care pathways, communication using the ISOBARNOW framework. There was also a focus on raising awareness about sepsis, recognition of signs of sepsis, online links to family stories, health service policies as well as additional resources. Successful completion of the online package was assessed using a multichoice quiz. Prior to Study III, there was a focused effort on raising staff awareness about sepsis and the sepsis escalation pathway through local presentations and distribution of lanyards. Prior to Study IV the package was further refined with expert input and review followed by testing in a clinical simulation environment. The education package development and evaluation has been reported in greater detail elsewhere.^27^

**Table 2:** Education and training strategies

Implementation preparation	Study I	Study II	Study III	Study IV
Website resources	x	x	x	x
Champions workshop	x	x	x	x
Support for champions	x	x	x	x
Resources to support family involvement (posters, pamphlets)	x	x	x	x
Staff education sessions	In-person	In-person and online	In-person and online	In-person and online
Education content		Expanded	Expanded	Tailored
Video		x	x	x
Online learning package with embedded videos and sepsis component			x	x
Assessment of learning			x	x
Additional sepsis awareness raising:				
Lanyards			x	x
Local presentations			x	

### Context – what influenced it (the intervention)?

Context covers anything external to the intervention that may act as a barrier or facilitator to implementation.^23^ The ESCALATION system^19^ was developed in an Australian state where unique healthcare challenges are faced due to geography, isolation, and sparse population,^28^ significant variations exist in healthcare services, including health professionals’ experience and expertise in caring for acutely unwell children.

For Study I the following data collection methods were used to assess feasibility, acceptability and identify contextual factors that may influence implementation:

- *Transfer audit* captured the characteristics of patients transported from participating sites to the ED at the specialist children’s hospital and documented communication about patients’ clinical condition. Eligible cases were identified through the children’s hospital ED information system.
- *Escalation of care audit* involved identifying patients with clinical deterioration whose care was escalated to urgency level of a Medical Emergency Review. This allowed examination of routinely collected data in the clinical deterioration context for patients unexpectedly transferred to higher level within a study site or to another hospital. Eligible cases were identified by the Champions and medical emergency response records.
- *Health Professionals Survey* adapted from two existing Australian surveys^26,29^ to understand health professionals’ attitudes to the intervention. There were 30 items using 5-point Likert-type agreement scale responses, five dichotomous responses, and one open-ended question. The survey was pre-tested for clarity with four expert nurses, with minor wording changes made.
- *Champions feedback*: Champions reported on feasibility and acceptability of education and training delivered, and reported any documented adverse events associated with the intervention.

### Mechanisms of impact - how does the delivered intervention produce change?

Mechanisms of impact involves understanding how the intervention produces change and was examined through participants’ responses and interactions with the intervention in context.^30^ Data collection methods were designed to assess acceptability of the intervention by understanding users’ (doctors, nurses and parents) attitudes.^22^ For reporting purposes, the definition of “parent” was mother, father, guardian or whoever identified as primary caregiver. We collected the following data:

- *Health Professionals Focus groups*^31^ were held in-person and online following completion of study periods. An adapted Claims, Concerns, Issues method^26^ was used to collaboratively explore; *Claims* by participants identifying what worked well?*, Concerns* by participants identifying what could be better?*, Issues* (solutions) were established by group consensus and action plans identified. Focus groups were conducted by FG (PhD), AC (Hons) and PF (Master), all Registered Nurses with experience conducting focus groups and interviews.
- *Parent interviews* For Study I, a convenience sample of parents were recruited while their child was an inpatient or contacted by telephone post-discharge. A semi-structured interview guide was informed by the health consumer advisory groups. The interview guide was pre-tested with two parents who were not study participants. Interviews were conducted in-person or by telephone. Interviews were audio-recorded with participant permission.
- For studies II–IV, to understand if the family involvement intervention component was delivered as intended, the bedside audit was expanded to include the following *Parent questions* (if a parent was present); “has your nurse asked you how you feel your child is doing?”, “has your nurse regularly included you in your child’s assessment?”, and “do you know about CARE Call?” (state-wide family escalation of care process).

Table 3 depicts indicators collected, noting the focus shift from initially being on improving and refining the intervention (Study I) to later more focused evaluation to promote implementation fidelity (Study IV).

**Table 3:** Feasibility and acceptability indicators

Indicators	Measures	Study I	Study II	Study III	Study IV
Implementation – What was delivered?	Number (percentage) of sites where intervention used	x	x	x	x
	Number (percentage) of patients where intervention used	x	x	x	x
	Bedside audit of documentation				
	• Number (percentage) clinician/ family concern was identified	x	x	x	x
Implementation preparation	Number (percentage) of Champions who attended workshop	x	x	x	x
	Number (percentage) of nurses and doctors received education and training	x	x	x	x
Context – what influenced it?	Escalation of care audit –number and characteristics of patients and escalation of care events	x			
	Transfer audit	x			
	Health Professionals survey	x			
	Champions reported documented adverse safety events	x	x	x	x
Mechanisms of impact - how does the delivered intervention produce change?	Health Professionals focus groups	x	x	x	x
	Parents interviews	x			
	Number (percentage) of parents were asked how their child was doing			x	x
	Number (percentage) of parents who reported being involvement in child’s assessment			x	x
	Number (percentage) of parents who were aware of the family escalation of care process			x	x

## Data analysis

Quantitative data were collated descriptively. Survey open-ended questions were analysed using summative content analysis.^32^ Focus group data were analysed in real-time by two female researchers (FG[RN, PhD], AC[RN, BN], or PF[RN, MN]) with participants to ensure all issues were confirmed and clarified. Issues were then prioritised and used to develop action plans. The contributions of all groups were collated, findings themed and shared with a selection of participants for verification.^31^ Parent interview data were collected and analysed iteratively so that themes identified in early interviews were explored in later interviews. A structured multi-step process for inductive interview thematic analysis^33^ was followed. A second research team member checked data coding. An audit trail of decisions was retained. The health consumer advisory groups provided feedback on findings. The mixed methods analysis included data integration of the quantitative and qualitative findings using joint displays^34^ as a structure combining the Claims, Concerns, Issues^31^ approach with the MRC framework^23^ concepts to understand what and how the intervention was delivered, contextual factors and mechanisms of impact. Our interpretation of the findings from multiple data sources was verified with the steering group.

Ethical approvals were obtained from Health Services RGS 3192 and 1940, University (HRE2019-6412) Human Research Ethics Committees and West Australian Aboriginal Health Ethics Committee (951). For reporting qualitative research the Consolidated Criteria for Reporting Qualitative Research reporting checklist was followed.^35^ For this article we reported on the implementation component of the Standards for Reporting Implementation Studies (StaRI) checklist.^36^

## Results

### Implementation – what and how the intervention was delivered

At participating sites, during the four studies, the ESCALATION system was used in place of existing paediatric EWS for all paediatric patients (age 0–16 years) who presented to the ED and or were admitted. For Hospital A (specialist paediatric hospital): Study I one ward, Study II two wards, Study III all inpatient areas; and for Hospitals B-E (hospitals with or without specialist paediatric services) ED and inpatient areas where paediatric patients were cared for (Table 4).

**Table 4:** Results feasibility and acceptability indicators

Characteristics	Study I Hospitals A-F	Study II Hospital A & E	Study III Hospital A & E	Study IV Hospital C
*Implementation – what was delivered*				
Sites	6	2	2	1
Patients	15,536 (100%)	8611 (100%)	18,437 (100%)	4866 (100%)
Number of bedside audits	249	123	250	30
Clinician/family concern documented	36 (14.5%)	7(6%)	49 (20%)	20 (66%)
*Implementation – how intervention was delivered*				
Champions attended workshop	18	10	17	5
Health Professionals received education and training	362/1202 (30%)	200/245 (82%)	707/871 (81%)	408/466 (87%)
*Context*				
Transfer audit	151			
Escalation of care audit	11			
Health Professionals survey responses	205/1202 (17%)			
Female	177 (88%)			
Nurses	190 (94%)			
Doctors	10 (5%)			
ED staff	129 (64%)			
Ward staff	172 (36%) (*Missing 5)*			
Champions feedback	No adverse events reported	No adverse events reported	No adverse events reported	No adverse events reported
*Mechanism of impact*				
Health Professionals focus groups:	14	8	9	2
Nurse participants	70	21	42	10
Doctor participants	5	n/a	n/a	6
Parent interviews (Mothers/Fathers)	13 (11/2)			
English as first language	13			
Aboriginal	1			
ELD background	1			
Number of hospital admissions for child:				
1	7			
>1	6			
>8	2			
Bedside audits – family involvement:				
Parents were asked how their child was doing			74/82 (90%)	25 (100%)
Parents were included in child’s assessment			70 (85%)	25 (100%)
Parents were aware of the family escalation of care process			50 (61%)	10 (40%)

*ELD* Ethno-linguistically diverse

The bedside audits demonstrated chart documentation was used as intended; assessed by key safety indicators of correct chart for age range, recording date, time, clinical variables, calculated early warning score, escalation of care pathway utilised. Examining routinely collected data revealed a policy-practice gap for patient monitoring and documentation by nurses. For example, for Study I patient blood pressure at ED presentation or ward admission was recorded on 167 (67%) charts. Additionally, gaps were identified in nurses’ knowledge and skills related to clinical assessment during the training sessions. Following targeted education, audit and feedback, the frequency of documented blood pressure measurement increased over subsequent studies but remained the most common missing variable. During focus group discussions many nurses reported they did not routinely measure children’s blood pressure and requested further clarification about when measuring blood pressure was appropriate. Over the four studies, family or clinician concern was recorded for between 6 and 66% of charts. The high proportion of family or clinician concern captured in Study IV was explored and found to be related to misinterpretation of the variable by staff, and targeted education was provided. See supplementary file Bedside Audit Study 1.

All Champions attended workshops as planned. Study I evaluation showed the Champions were satisfied with the preparation received and resources. They did report being unable to reach all health professionals to provide education and training at their sites, particularly in ED. In Study 1 only 362 (30%) health professionals received education across the six sites reflecting it had not been feasible to deliver in-person training as planned. This was backed up by Study I staff survey responses and focus groups findings. As a result, we set and achieved a target of 80% of health professionals to receive education and training using multiple delivery approaches in preparation for Studies II–IV.

### Context - what influenced it?

As previously described, six hospitals were purposively selected for Study 1 to enable assessment of feasibility across a range of settings reflecting a variety of health facilities and paediatric services, from the specialist children’s hospital to a small district hospital with no specialist paediatric service (Table 1).

#### Transfer audit

During Study I, 151 patients were transferred from participating sites to the specialist children’s hospital, with 141 patient health records available for review. Approximately 20% (30) patients were transferred for clinical deterioration. The audit showed the escalation process documentation provided a common language across sites.

#### Escalation of care audit

During Study I, the feasibility of the escalation of care process was assessed by examining health records of 11 patients identified as receiving a Medical Emergency Review. Six patients were successfully managed at their hospital. Five patients were subsequently transferred to the specialist children’s hospital. The audit showed the escalation process documentation provided a common language across sites.

#### Health professionals surveys

During Study I, surveys were used to collect feedback on attitudes towards the new system. Overall, there was strong agreement (81%) with the system design and features. The majority (68%) agreed the escalation pathway was appropriate for use at their hospital. Almost three quarters (71%) agreed the escalation pathway assisted them managing deteriorating patients. Some of the new system features initially received mixed responses. One half (50%) of participants agreed that the addition of a family concern variable assisted them to seek parents’ views about their child’s condition, 29% were neutral and 21% disagreed. Regarding the communication framework, 43% agreed the framework supported their escalation of care communication, while 30% were neutral and 22% disagreed. Disagree responses were related to lack of understanding by health professionals who had not received education and training.

#### Champions feedback

Champions reported no documented adverse patient safety events associated with the ESCALATION system use.

### Mechanism of impact - how does the delivered intervention produce change?

#### Health Professionals focus groups

For Study I and Study II, the purpose was to understand the acceptability of intervention delivery in each context, any unintended effects and identify any refinements required (Table 4). Themes developed from the Claims, Concerns, Issues process were; ‘supporting assessment’, ‘chart design’ and ‘implementation’.

At Study I, health professionals were positive about many system features that supported clinical assessment, including the integrated family concern variable. Health professionals who had not received education were less certain about its value. Most reported they had not purposefully used the ISOBAR NOW communication framework. Several nurses requested more education about paediatric clinical assessment. Nurses who were unfamiliar with using a scoring system requested more education and practice opportunity, and more local Champion support. Action plans included education and training content to be refined and expanded to explicitly address identified knowledge gaps. Health Professionals were positive about the overall chart design, especially the structured ABCDE format. Some features during development that had been considered essential initially, such as space to write initials and document modifications, were later found to be redundant. Despite the chart being A3 size, there remained a concern about the small font size and small space available for documentation. Feedback about being prepared for implementation was mixed. Those who had received education felt well prepared, whilst those who had little or no education were less confident. See Supplementary File: Focus Groups Study I and II key findings.

For Study III and Study IV, the purpose of focus groups was to evaluate the preparation for using the ESCALATION System, acceptability of additional features of Version 3.0 that included sepsis recognition prompts and sepsis escalation of care pathway, and collaboratively address any remaining concerns to promote intervention fidelity (Table 3). Health professionals reported they felt well prepared for implementation, despite timing coinciding with COVID-19 pandemic related high clinical activity. Most had completed the online education package, although they reported a strong preference for in-person education. Some nurses reported the sepsis education content was new information they had not previously known. A small number of medical staff held reservations about not including the temperature variable in the early warning score, although others appreciated how it reduced unnecessary escalation of care for children with fever. Further clarification was still needed about documentation expectations. Most reported they had not purposefully used the ISOBAR NOW communication framework. For chart design and new features, all were supportive of the addition of the sepsis escalation pathway. Some reported anecdotally, since implementation of the ESCALATION system, they felt patient deterioration was being detected sooner. Action plans included practical solutions for more easily locating and using the chart at patient bedsides, and further targeted education with audit and feedback to monitor behaviour change. See Online File: Study III and IV Claims, Concerns, Issues.

#### Parent interviews

During Study 1, 13 interviews were conducted with a convenience sample of parents of children who were inpatients. Six of the children had experienced a clinical deterioration event during their hospital stay (Table 4). Two themes were developed. Theme 1 Parents reported ‘Being included in their child’s assessment’ and how being invited to contribute was empowering: *“*I just used to never say anything because I’d worry they’d just shrug you off … so you feel like you have a bit more say” (P10). Theme 2 ‘Family involvement posters supported communication of concerns*’* about their child’s condition as “I found it a lot easier to describe what was going on” (P10). Key factors influencing parents’ involvement were how health professionals communicated with families (Table 5).

**Table 5:** Parent interviews themes and supporting quotes

Theme 1: Being included in their child’s assessment	Supporting quotes
Parents felt that nurses involved them in clinical assessment	They asked me my opinion about how well or unwell he was every time they came in to check him (P07) If I notice any changes, if I’m concerned about her to let the nurse know and then if I didn’t feel like I was being listened to or answered then I could speak to the shift supervisor (P10)
A mother further explained how being invited to contribute was empowering	It’s a good idea because… sometimes you can feel like you’re left out of the loop and your input isn’t [valued], because you’re not a doctor… I just used to never say anything because I’d worry they’d just shrug you off … so you feel like you have a bit more say(P10).
Communication styles used by health professionals were important enablers for parents to feel confident to raise concerns	I felt pretty comfortable speaking to the nurses and the doctors … I never felt like I wasn’t getting the answer… they weren’t pushing me off or making me feel like … an idiot (P4).
A mother, of a child with a chronic illness, provided her perspective of being an advocate, to be able to speak up to share her child’s complex medical history, especially during handover between health professionals who may be unfamiliar with the full details	What does his breathing normally sound like? Sometimes with handover it only takes for one little part of, especially with his history… to be told wrong and then it becomes a bit like the Chinese whisper and then the next person tells it a bit differently … by the time you come back the next day it’s like oh hang on actually no that’s not quite right (P3).
**Theme 2: Family involvement posters supported communication of concerns**	**Quotes**
Almost all parents recalled seeing posters	I just seen a poster that said if you feel concerned we will listen (P01) To be honest, I just wasn’t in the state of mind to actually… pay attention to those things (P11)
Most parents reported there was a lot of information to read on the poster, yet the content was helpful	… you kind of gloss over at first but after a while of looking at it you do kind of understand what it’s talking about … then again at [another hospital] they had it in the room which was you know pretty helpful … when you’ve got time you can sit there and read it…there is a fair bit of info on it. But it all made sense, it was all clear enough to follow (P4)
One mother described how she had used the poster information to explain her concerns	Cause sometimes you just know, you don’t feel like they’re right but it’s hard to voice what you think is going on. But when you read this [poster] I found it a lot easier to describe what was going on… like they aren’t weeing as much, and she is really tired. Rather than just … she doesn’t seem like herself, it was actually easy to describe what was going on (P10)
One mother indicated that she would like more detailed information about when to be concerned	I would have liked to know what the numbers meant a bit more and what was the safe range, because when it [monitor] would beep it would startle me in the room… it probably would have like put my mind at ease rather than me watching the monitor going oh is that a high number? (P07)
One mother (of twins) explained how at first she had questioned the congruency of the poster messaging ‘We are listening to you’ with health professionals’ behaviours. This mother’s actual experience in hospital and relationship with nurses caring for her babies was positive and reassured her.	I did [see the posters] to be honest but I didn’t know how truthful they are, so if I did say something will they actually listen?…for example, because I stood up for myself when I did say something will they be reluctant in treating one of the children if they needed treatment But to be honest with you I was wrong because when I did speak to the nurses, said I’m not happy … they actually did something about it (P06)

For both Study III and IV, based on the bedside audit results, over 85% of parents reported they had been asked for their opinion about their child’s condition and felt included in their child’s assessment. However, parent awareness about the family escalation of care processes remained low at 61% and 40% indicating a continuing need to raise awareness.

#### System refinements across Study 1 – Study IV

In-principle agreement had been reached to implement ESCALATION in all hospitals at the completion of Study I using Version 2.0. As the testing process evolved along with emerging evidence, it was agreed to add to the sepsis recognition prompts and include a sepsis escalation pathway into Version 3.0. It was also recognised that while the Version 3.0 escalation of care pathway was broadly supported, it would not fully meet the contextual needs of all users in terms of escalation of care responses available in settings other than the specialist children’s hospital. The process of revisions sequentially resulted in ESCALATION version 4.0 including one of three escalation pathways for; specialist children’s hospital, metropolitan hospitals, regional/rural hospitals. See Supplementary file. Version 4.0 PARROT chart age <3 months for Regional/Rural Hospitals. Further detail of the system refinements is presented in Table 6.

**Table 6:** System refinements Version 1.0 –> Version 4.0

Component refined	Version 1.0 - > 2.0	Version 2.0 - > 3.0	Version 3.0 - > 4.0
Education and training	Use of video Aim for 80% Health Professionals to receive training Increased site support Further Health Professionals education to understand the family concern variable	Minimum 80% Health Professionals attended education and training Increased use of active learning techniques Preparation using online package	
Chart layout	Orientation from portrait -> landscape	Decluttering redundant features Formatting	
Variables		Increase to 3 modifications Addition sepsis recognition prompts Removal signature key	
Parameters		Revised parameters for age ranges 5–11 and 12 plus Additional recording eg. mean blood pressure, blood glucose	
Escalation of care pathway		Addition sepsis escalation of care pathway	Change from 1 generic pathway to 3 pathways: Metropolitan Regional /Country Children’s
Family involvement poster/flyer	More visual, less wordy	More visual, less wordy	More visual, less wordy

## Discussion

The ESCALATION System was developed as a standardised evidence-based approach to facilitate early recognition and response to paediatric clinical deterioration. Encompassing integrated family involvement, sepsis recognition prompts and an escalation pathway with a supported communication framework, it is suitable for use in a variety of clinical settings. Whilst there was willingness by all stakeholders to adopt a uniform standardised paediatric EWS, for many health professionals, especially those unfamiliar with using scoring systems, adopting the ESCALATION system involved using a cognitively different approach to assessing clinical deterioration and necessitated greater change to existing behaviours.^37^

Implementation involves understanding and capturing whether the intervention was delivered as intended. Understanding adherence, and what was delivered in practice, helps distinguish between adaptions made to suit different contexts and changes made that may undermine implementation.^22,23^ Additionally, it is important to understand how the intervention was delivered to provide generalisable knowledge on how best to ultimately implement the strategy. Our focus was assessment of feasibility and acceptability to refine the intervention design^23^ and evaluate the education and training strategies for implementation.

Including the perspectives of diverse stakeholders and users (including health consumers with experience of being parents or patients in hospital) through meaningful engagement created the collaborative environment for iterative system refinements. For example, we had previously explored inclusion of a sepsis escalation pathway, but inclusion was not agreed to in earlier versions. As a result of ongoing stakeholder engagement, consensus was reached to include a sepsis escalation pathway in Version 3.0. This meant that when the ESCALATION system was fully implemented, it provided an integrated paediatric sepsis clinical support tool, coinciding with the release of the Australian Sepsis Clinical Care Standard requiring health services to use a clinical support mechanism to help recognise sepsis early and escalate care when required.^17^

The version for full implementation included tailoring of escalation pathway responses to match differently resourced hospital contexts. Our reflection accounting for the later version system changes was stakeholder buy-in and willingness to achieve a uniform approach was initially associated with temporary changes to health service policies and procedures managed during the research phases. The reality of full implementation required all hospitals and health services to undertake permanent policy and procedure changes. At a local level, this then involved a wider examination about how the ESCALATION system and pathway response could work in all hospital environments. The result is a highly refined ESCALATION system, fit for purpose, which is likely to be well accepted across a health system that has substantial contextual variation.

The ESCALATION system scoring represented a significant practice and behaviour change for many intended users across a variety of clinical settings. Change management research has shown that smaller differences between current work practices and new processes make implementation easier.^37^ We considered that optimising staff preparation for behaviour change to be a key step towards ensuring implementation success. A completion rate of 80% by health professionals for the online education package increased reach opportunity, and further supported site implementation success.

Through delivering education in preparation for implementation we identified gaps in nurses’ knowledge and skills related to clinical assessment of children, in particular uncertainty and conjecture about measuring blood pressure. Low rates of measuring blood pressure in children have long been reported^38,39^ Barriers described include concerns about measurement accuracy, lack of available equipment as well inability to remember acceptable parameters for different age ranges.^38^ We attempted to address knowledge and skill gaps in our context by refining the education content to include more explicit information and instruction for nurses who may be less confident caring for children.^40^ However, the reality of narrowing this theory-practice gap appears to be too complex to be resolved simply through in-service education and supply of equipment. This continues to be a conundrum for all paediatric EWS that include manual recording of variables.^39,41,42^

Our feasibility and acceptability assessment specifically focused on the new system feature of integrated family involvement through the clinician/family concern variable. Initially we found health professional attitudes varied across settings and appeared to be associated with whether individuals had received preparation. Attitudes towards the clinician/family concern variable became more favourable over time. This finding is reflected in the literature reporting increasingly more positive views of health professionals towards family and patient involvement in escalation of care^43^ and patient safety initiatives.^44^ The experiences of families strongly supported integrated family involvement in the ESCALATION system and highlighted for the system to work as intended, effective communication between health professionals and families in hospital is key.

The major strengths of this study were the systematic and theoretically informed approach, sustained stakeholder engagement and diverse data sources collected across several hospitals over four time periods enabling refinements to be made iteratively to suit contextual variations. There are several study limitations to acknowledge. The evaluation of families’ experience of involvement was limited to a small number of participants who were English speaking and health literate. Our findings may not reflect views or experiences of families with lower health literacy or low language proficiency; known risk factors associated with patient safety incidents in hospital.^45^ Specifically, we did not examine the influence of cultural and linguistic diversity on families’ capacity to be involved. The willingness and ability of families to be involved in assessing their child, recognising changes and escalating care is underpinned by assumptions that families can recognise clinical deterioration and feel safe, confident and adequately prepared to raise their concerns. Health consumers from culturally and linguistically diverse backgrounds have reported having little experience questioning doctors or shared decision making in healthcare.^46^ Not yet fully understood is the magnitude of the impact of cultural and language barriers, low health literacy, power imbalance and low level of cultural safety experienced in the hospital environment.^13,47^ We previously reported on family involvement in escalation of care for Aboriginal children in hospital, finding that more effective, culturally relevant communication by health professionals is needed.^48^ Further research is required to understand how to effectively enable the most vulnerable families in hospital to be involved in recognising and responding to changes to their child’s condition.

The components of the ESCALATION system included the structured communication ISOBAR NOW. Whilst feedback from health professionals was positive about ISOBAR NOW, it was evident this component of the intervention was not used fully as intended. The ESCALATION system, a complex intervention, is composed of several interacting components where implementation involved substantial cognitive, behaviour and practice changes. At the time of implementation, equal emphasis was not placed on the communication component and proved insufficient to achieve cultural change. Fully implementing the communication elements of the ESCALATION system in the future will require a targeted approach.

## Conclusion

The standardised evidence-based ESCALATION system is designed to facilitate early recognition and response to paediatric clinical deterioration with integrated family involvement, sepsis recognition prompts and an escalation pathway with a supported communication framework. Our series of studies was conducted over four time periods across several hospitals, each with contextual variations within a large Australian state. We systematically assessed feasibility, acceptability and examined concepts of Implementation, Context and Mechanisms of Impact from the perspectives of users. Iteratively refining the system in this way enabled development of an acceptable and feasible intervention ready for full-service implementation. Our education preparation package targeted identified gaps in nurses’ paediatric clinical assessment knowledge and practices. More targeted work will be required to support full use of the communication framework component. Further research is required to understand how best to support all families to be involved in recognising and responding to changes in their child’s condition. Our approach highlighted the importance of adequate preparation for complex intervention implementation. Continuing intervention effectiveness and long-term sustainability will require ongoing monitoring and evaluation, often overlooked in implementation research.

The data that support the findings of this study are available from Fenella Gill but restrictions apply to the availability of these data. The Human Research Ethics Committee did not approve the data to be publicly available. Data are however available from the authors upon reasonable request and with permission of Fenella Gill.

## Supplementary information

supplementary file

supplementary file
