# Supplementary material for: Feasibility and acceptability of implementing an evidence-based ESCALATION system for paediatric clinical deterioration
Source: Pediatr Res. 2024 Aug 13;97(3):1047–57. doi: 10.1038/s41390-024-03459-y (PMC12055582; doi:10.1038/s41390-024-03459-y)
Supplement: Supplementary file 1 — supplementary file [file 41390_2024_3459_MOESM1_ESM.pdf]

Online supplementary file **Bedside audit Study 1**

1. Site ___________________________________

2. Department(s) ___________________

3. Patient age group chart

☐ <3 months
☐ 3-12 months
☐ 1-4 years
☐ 5-11 years
☐ 12 years and over

4. Was the correct age chart used for the patient?

☐ Yes, ☐ No

5. Was the date for the observations recorded?

☐ Yes, ☐ No

6. Were the times for the observations recorded?

☐ Yes, ☐ No

7. Was there a written monitoring plan for this patient?

☐ Yes, ☐ No

8. Were the core observations measured according to the monitoring plan?

☐ Yes, ☐ No

9. How many sets of observations were recorded in the last 24 hours?

10. How many sets of observations were recorded in the last 48 hours?

11. Required frequency of observations as documented in the monitoring plan

☐ 1 hourly
☐ 2 hourly
☐ 4 hourly
☐ 8 hourly

12. Were there any core observations missing as per the monitoring plan in the last 24 hours?

☐ Yes, ☐ No

13. If yes, which observations were missing?

☐ Respiratory rate
☐ Respiratory
☐ Oxygen therapy
☐ Oxygen saturations
☐ Heart rate
☐ Capillary refill time
☐ Blood pressure

- AVPU
- Temperature
- Pain score

14. Were there any modifications?

- Yes, - No

15. If yes, were these modifications correctly written?

- Yes, - No

16. Indicate which modifications were made

- Respiratory rate
- Oxygen saturation
- Heart rate
- Blood pressure

17. Were the nurse initials completed for each set of observation in the last 24 hours?

- Yes, - No

18. Was the scoring system used on this chart?

- Yes, - No

19. If yes, was the score added up correctly?

- Yes, - No

20. How many triggers occurred in the last 24 hours?

- 1
- 2
- 3

21. For the last score triggered, were total observations completed?

- Yes, - No

22. Did the patient's condition meet any criteria for escalation in the last 24 hours?

- Yes, - No - unsure

23. If yes, which escalation of care was indicated?

- Senior nurse
- RMO or equivalent
- Rego or equivantl
- Consultant
- MET/ Code Blue Medical emergency

24. If yes, was care escalated according to the escalation plan?

- Yes, - No - unsure

25. Was a clinical intervention and escalation of care documented on the chart?

- Yes, - No

26. Was the clinician/ family variable documented on the chart?

- Yes, - No

27. If yes, who indicated their concern?

- Family

- ☐ Nurse
- ☐ Medical

Adapted from the Audit Tools and Quality Measures for Recognition and Response Systems: Audit and Evaluation Tools available on the Australian Commission of Safety and Quality in Health Care, located **https://www.safetyandquality.gov.au/our-work/recognising-and-responding-to-clinical-deterioration/evaluating-recognition-and-response-systems/quality-measures-for-recognition-and-response-systems/**
