# Supplementary material for: Feasibility and acceptability of implementing an evidence-based ESCALATION system for paediatric clinical deterioration
Source: Pediatr Res. 2024 Aug 13;97(3):1047–57. doi: 10.1038/s41390-024-03459-y (PMC12055582; doi:10.1038/s41390-024-03459-y)
Supplement: Supplementary file 2 — supplementary file [file 41390_2024_3459_MOESM2_ESM.pdf]

Online supplementary file: Focus Groups Study I and II key findings

Themes		Study I	Study II
Supporting assessment	Claims	Uniform system<br>5 charts<br>Visual cues<br>Standardised assessment<br>Weighted and unweighted parameters<br>Including family	Age- appropriate components (including pain scales)<br>Less need for modifications<br>Standardised assessment<br>Weighted and unweighted parameters<br>Family /clinician concern supported escalation of care communication
	Concerns	Uncertainty about value add of family/clinician concern	Communication framework not used<br>Uncertainty when to write numerical values<br>Revise some parameters (age 12yrs+)
Chart design	Claims	ABCDE format logical<br>Escalation pathway appropriate<br>iSOBAR NOW communication framework useful	ABCDE format easy to use<br>Space for numerical values is an improvement
	Concerns	Some redundant features<br>Additional features requested<br>Parameters refinement required<br>Improvement to formatting required<br>ISOBAR NOW communication framework not used	Some features not used (signature key)<br>Improvement to formatting required (remains too small)<br><br>Further additional features requested
Implementation	Claims	Positive about practice change<br>Had been sufficiently prepared	Preferred in-person education<br>Practice using scenarios
	Concerns	Not all had received education and training<br>Identified deficit in nurses' knowledge<br>Unfamiliarity with scoring system<br>Pilot period too short	In-person education preferred<br>First two weeks were challenging<br>More education about clinician/family concern requested<br>More education and support requested<br>Video too long<br>Family poster not displayed in all areas
	Issues	Revise chart to improve usability<br>Expand education and training<br>Consider different learning needs	Revise chart to improve usability<br>In-person education to include clinical scenarios<br>Tailored education required for doctors<br>Audits and feedback recommended

Focus Groups Study III and IV key findings

Themes		Study III	Study IV
Preparedness	Claims	Felt well prepared<br>Having local staff development nurses and researchers available<br>Felt best prepared out of three concurrent major practice changes	Felt well prepared although we are still transitioning to full use<br>Intervention has been a positive initiative
	Concerns	Too many practice changes occurring at the same time<br>Overwhelmed – staff shortages and COVID-19 activities	Nil
	Issues		Follow up audits to monitor full use
Education	Claims	Positive about sepsis module	
	Concerns	In-person education preferred<br>Little previous sepsis education (nurses)<br>COVID-19 restrictions limited in-person education	Temperature variable no longer contributes to early warning score<br>Focus has been on chart documentation – uncertainty about when to write numerical values<br>ISOBAR NOW communication framework not being used
	Issues		More education required to support using ISOBAR NOW communication framework
Chart design (Version 3.0)	Claims	Clinician/family concern<br>Pain scores<br>Sepsis recognition prompts	Clinician/family concern<br>Respiratory distress scale<br>There is now less missed deterioration<br>A-E structure<br>Removal of temperature as weighted variable<br>Sepsis recognition prompts
	Concerns	Still getting used to new chart<br>Some concern about temperature not contributing to score	Chart is complex<br>Portrait orientation inconvenient – prefer landscape<br>Sedation scoring is doubled up with current ED sedation chart. (Changes underway to rationalise)<br>Family concern can reflect longer ED wait time<br>Pain assessment FPS-R not preferred<br>ISOBAR NOW communication framework not being used
	Issues	Recommended clinical simulation, active learning, refresher education, more sepsis education,<br>Audit and feedback to target behaviour change to measure and document observations	Trial using A3 clip chart (for portrait orientation)<br>To clarify write numerical values in addition to graphical when value is abnormal<br>Reinforce join up dots for graphical display
